# Children’s psychological perceptions and responses to pain and fear during venipuncture: a qualitative study using an arts-based data collection method

**DOI:** 10.1007/s00431-026-06962-y

**Published:** 2026-04-30

**Authors:** Sherzad Khudeida Suleman, Nizer Yahya, Stefan Nilsson, Karin Enskär

**Affiliations:** 1 Department of Women’s and Children’s Health, Uppsala University, Uppsala, Sweden; 2 Pediatric Medicine Unit, College of Medicine, Duhok University, Duhok, Kurdistan Region Iraq; 3 Institute of Health and Care Sciences, Sahlgrenska Academy, University of Gothenburg, Gothenburg, Sweden; 4 Pediatric Psychiatric Nursing Department, College of Nursing, University of Duhok, Duhok, Iraq

**Keywords:** Venipuncture, Psychological experiences, Art-based intervention, Drawing and Storytelling, Qualitative study

## Abstract

Venipuncture procedures can be highly distressing for paediatric patients, potentially leading to short-term and long-term negative effects on their well-being and future medical experiences. This study aimed to provide a rich description of children’s psychological experiences during venipuncture procedures within a specific cultural context. A qualitative descriptive design was employed. Fourteen paediatric patients aged 6–12 years admitted to Heevi Pediatric Hospital’s departments in the Duhok province of the Kurdistan Region of Iraq participated in the study. Data were collected using the Drawing and Storytelling (DS) technique and analyzed through thematic analysis. Thematic analysis revealed three primary themes: (1) pain and fear during venipuncture, (2) parental support as a psychological buffer, and (3) ease and comfort strategies. Findings highlight the importance of child-centered, family-inclusive approaches to improve psychological well-being during medical procedures.

*Conclusion*:Paediatric patients experience pain and fear during venipuncture, but also feel pride and relief. Parental support and strategies like preparation and distraction help reduce anxiety. Implementing child-centered and family-inclusive care, informed by understanding children’s unique perspective and family dynamic, is crucial for improving psychological well-being during invasive medical procedures.

**Table:** 

**What is Known:** • *Venipuncture causes significant procedural pain and fear in children, with poorly managed distress linked to short-and long-term psychological consequences*. • *Parental presence and psychological interventions such as preparation, distraction, and praise are established strategies for reducing pediatric procedural distress*.
**What is New:** • *This is the first qualitative, arts-based study using the Drawing and Storytelling technique to explore venipuncture experiences among Kurdish children in the Iraqi Kurdistan Region*. • *Parental support operates as a dynamic, bidirectional psychological buffer within the family system, and children themselves validate comfort strategies within this culturally underrepresented context.*.

**What is Known:**

• *Venipuncture causes significant procedural pain and fear in children, with poorly managed distress linked to short-and long-term psychological consequences*.

• *Parental presence and psychological interventions such as preparation, distraction, and praise are established strategies for reducing pediatric procedural distress*.

**What is New:**

• *This is the first qualitative, arts-based study using the Drawing and Storytelling technique to explore venipuncture experiences among Kurdish children in the Iraqi Kurdistan Region*.

• *Parental support operates as a dynamic, bidirectional psychological buffer within the family system, and children themselves validate comfort strategies within this culturally underrepresented context.*.

## Introduction

Venipuncture is a common yet distressing procedure for paediatric patients, often causing procedural pain and fear with significant psychological consequences[1, 2]. The term “psychological experiences” is used throughout this paper to specifically denote the affective and cognitive dimensions—such as fear, anxiety, anticipation, and perceived pain—that constitute the child’s subjective reality during the procedure [3]. While evidence-based interventions exist to manage procedural distress, few studies have explored children’s experiences in non-Western settings, particularly within the Iraqi Kurdistan Region, where cultural, contextual, and healthcare system factors may meaningfully shape how children perceive and respond to painful procedures.

Pain and fear during medical procedures are subjective experiences influenced by a child’s developmental stage, previous medical encounters, and the broader socio-cultural context. The perception and expression of these experiences are shaped by a complex interplay of biopsychosocial factors, including temperament, parental anxiety, and the healthcare environment, all of which are key contributors to anticipatory distress [4, 5]. Age, while often used as a proxy for development, should therefore be considered only a rough estimate within this multifaceted context [4, 5]. Anxiety is closely related to pain perception, and procedural distress often manifests as a combination of anxiety and pain that can be behaviourally indistinguishable [1]. Poorly managed pain and fear during venipuncture can carry both short-term consequences—including anxiety, avoidance behaviours, and somatic symptoms—and long-term effects such as increased pain sensitivity, heightened anxiety, and avoidance of medical care in adulthood, all of which can prolong and complicate subsequent healthcare encounters [1, 6]. Because memory is an active process, a stressful or painful procedure can further reinforce negative expectations that influence future procedural experiences. The caregiver’s role in managing and reducing children’s pain and fear during painful procedures is therefore significant; caregivers who themselves experience fear and stress in response to their child’s procedure may, in turn, amplify the child’s own pain perception [7].

This study is theoretically informed by two complementary frameworks: Child-Centered Care (CCC) and Family Systems Theory (FST). CCC provides the ethical and practical framework for prioritizing the child’s voice, agency, and individual needs within healthcare encounters [8]. Family-Centered Care (FCC) is a widely adopted model in paediatric healthcare that recognizes the family as an integral partner in the planning, delivery, and evaluation of care [9, 10]. To deepen the analysis of the familial dynamics identified by the children in this study, we additionally draw upon FST. FST posits that family members are emotionally interconnected, with each member’s behaviour and emotions affecting the others within the system [11, 12]. Contemporary applications of FST in paediatric settings highlight how parental anxiety can directly influence a child’s procedural distress, and conversely, how a child’s coping can affect parental stress [13]. This theoretical lens moves beyond simply noting parental presence to analyzing the dynamic, reciprocal interactions that constitute “support”, thereby complementing CCC and offering a more nuanced explanatory framework for family dynamics than the broader practice model of FCC alone [13, 14].

Although many evidence-based pharmacological (e.g. topical anesthetic) and psychological (e.g. hypnosis, distraction) interventions are available to manage pain and distress in children [15, 16], understanding how children themselves perceive and narrate these experiences remains essential to informing effective, contextually appropriate care. Understanding children’s views of the role of caregivers and nurses in managing procedural pain is particularly important. Anecdotal evidence suggests that the majority of nurses in Iraqi Kurdistan have not received updated training in paediatric painful procedures such as venipuncture, cannulation, or immunization, nor in the emotional and behavioural impact of these procedures on children [17]. This underscores the significance of continuing research in this setting to provide nurses with a more nuanced understanding of how these experiences manifest, and how care may be improved accordingly.

Several empirical research works have noted a growing recognition of the importance of the child’s perspective in healthcare research [18–20]. Paediatric researchers have increasingly acknowledged that children are capable of contributing valuable information about their own experiences, and participatory research approaches that position children as active contributors have gained considerable traction [21]. Culture can further impact the attitudes, expressions, and behaviours of children towards pain and discomfort during invasive procedures [22], and attending to children’s cultural and social context is a defining feature of child-centered care [8]. While pain, fear, and procedural stress have been investigated predominantly from the perspectives of adults [23, 24], and the limited studies utilizing drawing-based techniques have been conducted almost exclusively in Western contexts [25, 26], literature examining the perceptions of children themselves—and particularly those from non-Western cultural backgrounds—remains scarce and warrants further development.

Therefore, the primary aim of this study was to explore and describe the psychological experiences of Kurdish children aged 6–12 years undergoing venipuncture, using the Drawing and Storytelling (DS) arts–based technique.

## Methods

### Study design

This study employed a qualitative descriptive design using arts-based data collection (Drawing and Storytelling—DS) to explore children’s psychological experiences during venipuncture. The qualitative descriptive approach was chosen to provide a rich, detailed understanding of the children’s lived experiences in a naturalistic setting, without imposing theoretical frameworks allowing participants’ perspectives to emerge directly from the data.

### Setting and participants

The study was conducted at Heevi Pediatric Teaching Hospital, a tertiary-care facility in the Kurdistan Region of Iraq, between June and November 2022. Participants were children admitted to various hospital units (e.g. emergency, medical/surgical wards) who underwent venipuncture procedures.

### Sampling strategy

Participants were recruited using consecutive sampling, a non-probability technique where all eligible, accessible children meeting inclusion criteria were invited to participate over the study period [27]. While often practical in clinical settings, consecutive sampling aims to capture all available eligible individuals during the recruitment window, offering a snapshot of the accessible population rather than being strictly purposive sampling which selects participants for specific characteristics. This approach was deemed the most feasible method to achieve a sufficiently varied sample within the hospital context during the study timeframe.

### Inclusion and exclusion criteria

#### Inclusion criteria

- Age 6–12 years.
- Fluency in Kurdish or Arabic.
- Scheduled for venipuncture.

#### Exclusion criteria

- Unstable medical conditions posing risks during participation.
- Communication/visual/motor impairments hindering expression or drawing ability.
- Refusal to participate.

This age range was chosen to ensure participants had sufficient cognitive and communicative abilities to engage in the drawing and storytelling activities. Language fluency was necessary for clear communication. Exclusion criteria were implemented to ensure participant safety and well-being, as well as to maintain the integrity of the data collection process.

### Data collection

Arts-based interventions, such as the Drawing and Storytelling (DS) technique, facilitate children’s expression of their impressions and reactions to painful procedures in ways that may not be thoroughly articulated through verbal communication. The data collection approach utilized in the current study is grounded in this methodological premise. While drawings are increasingly recognized as valuable tools for accessing children’s perspectives in research [28, 29], understanding these drawings without children’s input may present challenges due to potential inconsistencies or ambiguities in the visual representations, which could result in misunderstandings or, in more severe cases, a complete misinterpretation of the child’s intended meaning. To mitigate this limitation, we have adopted the DS approach, which is closely aligned with the established Draw, Write, and Tell (DWT) method [29]. The DWT approach allows children to depict their experiences through a created image and subsequently narrate their experiences in response to interview prompts, thereby ensuring that researchers do not misconstrue the meanings of the visual content[30]. Importantly, no prior research has explored the use of arts-based methods, specifically DS, as a data collection technique to understand children’s pain and anxiety during venipuncture procedures within this study’s cultural context, further justifying this methodological choice.

Following the recruitment and consent procedures (refer to Sampling Strategy and Inclusion and Exclusion Criteria), the researcher approached eligible children and their caregivers, providing verbal information regarding the study’s purpose and procedures. Written informed consent was obtained from parents or legal guardians, and verbal assent was confirmed with each child prior to participation. To ensure confidentiality throughout the study, pseudonyms were assigned to all participants.

Individual, face-to-face, semi-structured interviews were conducted following venipuncture to prevent any interference with the clinical procedure. These interviews lasted between 30 and 60 min, were audio-recorded with participants’ consent, and were subsequently transcribed verbatim.

The Drawing Story (DS) technique was utilized as the primary data collection method. This arts-based, child-centered approach involves children first creating a drawing in response to a guided prompt, followed by narrating the story of their artwork. In this study, the oral storytelling component was emphasized over written narration to better accommodate the developmental range of participants (aged 6–12 years) and the constraints of the clinical setting, thereby enabling children to articulate their drawings fluently and minimizing the risk of misinterpretation of their visual representations. In practice, children were provided with art materials (coloured pencils, markers, and paper) and invited to depict their experiences during the venipuncture procedure. Open-ended prompts guided the process, such as “Can you draw how you felt when the nurse was putting the needle in your arm?” and “What did you think about during the procedure?” Upon completion of their drawings, children were encouraged to narrate their artwork, elucidating what they had drawn and how it reflected their feelings and experiences. This multimodal approach facilitated children’s expression of emotions both visually and verbally, yielding a richer and more nuanced understanding of their psychological experiences than either modality could provide independently.

A semi-structured interview guide ensured the consistent administration of standardized questions across all interviews, addressing key topics including procedural understanding, emotional responses, coping strategies, and perceptions of parental and nursing roles. All drawings were digitized, and all data were anonymized and stored in encrypted files in accordance with institutional data governance requirements.

### Data analysis

Recruitment continued until the research team determined that the collected data provided sufficient depth and information power to address the research aim, and that ongoing analysis yielded rich, textured themes relevant to the study’s focus[31]. This judgement was guided by the principle of information power[33], which holds that sample size adequacy in qualitative research is determined not by numerical thresholds but by the richness and relevance of the data in relation to the study’s purpose—rather than by the attainment of a predefined saturation criterion [31].

Thematic analysis, following Braun and Clarke [32] six-step process, was used to synthesize data from interviews and drawings. Audio recordings were transcribed verbatim, and drawings were digitized. The analysis of drawings was integrated with the verbal narratives. No formal, standalone framework for analyzing children’s drawings was employed; instead, the children’s own explanations from the storytelling phase were used as the primary lens to interpret the visual content, ensuring the analysis remained grounded in the participants’ intended meanings. Visual elements (e.g. colour, size, composition) were noted as supporting evidence for the themes derived from the full dataset. Two independent researchers conducted open coding to identify key concepts, which were grouped into themes through iterative discussion. A thematic map visually represented relationships between codes (e.g. “needle size exaggeration”, “praise from nurses”) and themes (e.g. “Pain and Fear”, “Ease Strategies”).

Themes were reviewed for internal homogeneity and external heterogeneity. An age-based comparison explored potential developmental differences between younger (6–8 years) and older (9–12 years) participants. Visual analysis of the drawings—including the use of red and black colouring to signify pain and the exaggeration of needle size—was integrated with the verbal data to enrich interpretive depth. Inter-rater reliability was assessed using Cohen’s kappa (*κ* = 0.85), indicating excellent agreement between coders. Remaining discrepancies were resolved through peer debriefing.

### Researcher reflexivity

This study was conducted in Kurdistan as part of a transnational research collaboration. The team comprised the primary researcher (S.K.S.), a Kurdish-speaking paediatric nurse with clinical experience in the region; the local supervisor (N.Y.), who is also Kurdish-speaking; and two main supervisors (K.E., S.N.), who are not Kurdish speakers. This structure was intentionally designed to balance insider cultural and linguistic rapport with external methodological rigour. While the shared cultural positioning of S.K.S. and N.Y. facilitated trust and nuanced data collection, the potential for preconception was actively mitigated through regular peer debriefing sessions with the full supervisory team. The external perspectives of K.E. and S.N. served as a critical counterbalance, challenging assumptions and ensuring that the analysis remained anchored in the children's expressed perspectives, thereby enhancing the study's confirmability..

### Trustworthiness

#### Trustworthiness was ensured using criteria adapted from Shenton [33]

*Credibility* was supported by prolonged engagement, method triangulation (drawings and stories), and peer debriefing. *Transferability* is enabled through thick description. *Dependability and confirmability* were strengthened through a rigourous, collaborative analytic process involving two researchers in initial independent coding, followed by discussion to reach consensus on themes, supported by a detailed audit trail documenting all analytical decisions.

## Results

We conducted a thematic analysis on data collected from 14 child participants, ranging in age from 6 to 12 years old. Detailed participant characteristics are summarized in Table 1, providing important context for the thematic findings presented below. Through this analysis, we identified several key themes that shed light on the children's psychological experiences related to the venipuncture procedure.

**Table 1:** Participant characteristics and procedural context (*N* = 14)

Participants	Age (years)	Gender	Primary reason for admission
P1	8	Female	Respiratory infection
P2	12	Male	Respiratory infection
P3	11	Female	Urinary Tract Infection
P4	12	Female	Gastroenteritis
P5	8	Male	Gastroenteritis
P6	9	Female	Asthma Exacerbation
P7	10	Female	Diabetes Management
P8	7	Male	Febrile Illness
P9	9	Male	Respiratory infection
P10	10	Male	Gastroenteritis
P11	7	Female	Respiratory infection
P12	11	Male	Tonsillitis
P13	6	Female	Tonsillitis
P14	6	Male	Gastroenteritis

Two male participants of the same age group have been assigned sequential codes (P8 and P9) for distinction throughout the manuscript. Distraction techniques (e.g. conversation, guided imagery) were variably employed by some parents as part of routine care, but their specific use was not systematically documented per procedure

### Drawing

All 14 participants created drawings and shared stories about their venipuncture experiences, and their narratives were included in the full analysis. For illustrative purposes, a selection of nine drawings that vividly capture the key themes and variations is presented here. Every child included a depiction of themselves in their drawings. Twelve drawings featured the child alone, while two included the child’s primary caregiver. Only one child incorporated a healthcare professional (a nurse) into their drawing.

### Themes

#### Theme 1: Pain and fear during venipuncture

This theme explores the multifaceted nature of the children’s experiences of pain and fear during venipuncture, including their perceptions and expressions of pain, their feelings of fear, and any positive outcomes they derive from the experience. Understanding these aspects is crucial for comprehending the emotional and cognitive responses of children to medical procedures.

##### Subtheme 1.1: Perceiving pain

The children expressed their pain during venipuncture in vivid and emotive ways, reflecting the highly subjective nature of their experiences. Their descriptions varied widely, with some describing the pain as intense and others finding it more manageable than expected. These differences highlight how individual perceptions and expectations play a significant role in shaping their experience of pain. Notably, the visual modality of the DS method revealed an additional layer of this pain perception: children frequently used red or black colours in their drawings to highlight areas where they felt pain, with darker shades often indicating higher intensity. This chromatic strategy functioned as a non-verbal pain communication tool, allowing children to represent the severity of their experience through colour in ways that complemented, and at times exceeded, their verbal descriptions.

**Fig. 1:**
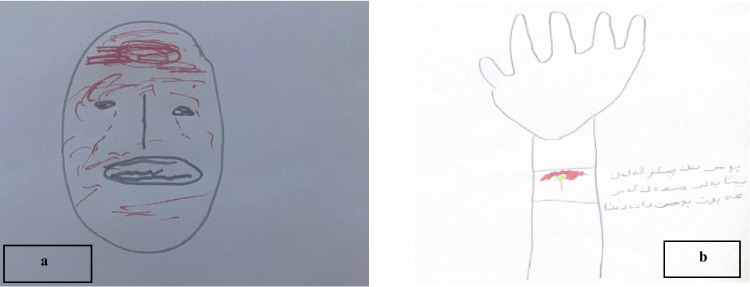
Drawings **a** and **b** depicting subjective pain experiences during venipuncture

“*It was very painful*”, stated an 8-year-old girl (P1) (see Fig. 1a), who selected the most distressed face on the Faces Pain Rating Scale, indicating severe pain. The expressive power of this self-report was mirrored in her drawing, in which she used bold red strokes to depict her arm, explaining, *“It hurt a lot here, so I made it red”*—a vivid illustration of how colour functioned as a direct proxy for pain intensity. In contrast, a 12-year-old boy (P2) reported, *“It was stinging but bearable”* (see Fig. 1b), indicating a more manageable level of discomfort.

##### Subtheme 1.2: Expressing fear

Children expressed that fear is a significant psychological response during venipuncture, extending far beyond the physical sensation of pain. This fear is deeply tied to the anticipation and uncertainty of the procedure, often more than the procedure itself. In their drawings, children often emphasized the size of needles and syringes, portraying them as larger and more intimidating than they are in reality. This exaggeration reflects how their imagination amplifies their fears, making the experience seem more daunting than it actually is. Beyond needle exaggeration, the spatial content of several drawings further illuminated the nature of children’s procedural fear. Two children focused specifically on the location of the venipuncture site itself, rendering the moment of needle entry with striking precision. The drawings of three children also incorporated the hospital environment, frequently portraying it as a large and imposing building—a representational choice that reflects how the institutional healthcare setting can itself become a source of fear and psychological threat for young patients.

**Fig. 2:**
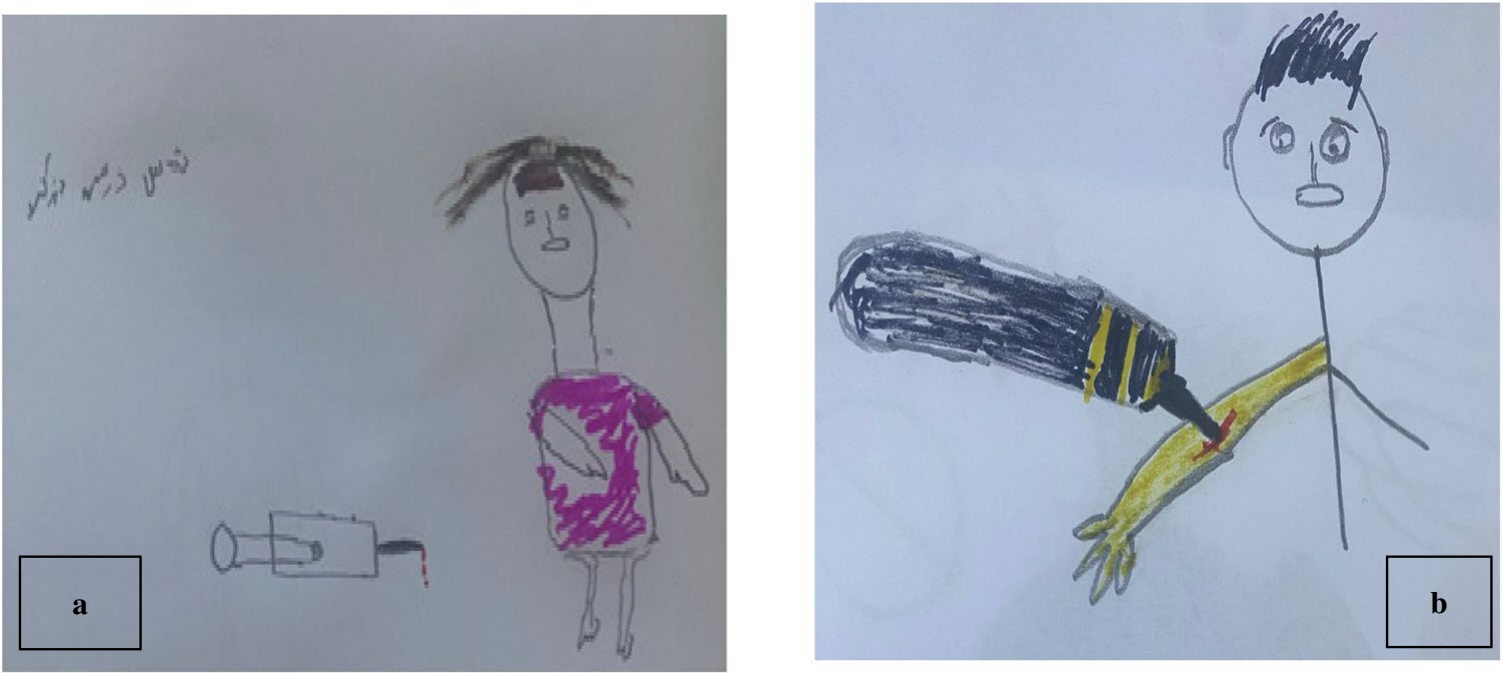
Drawings **a** and **b** depict children’s visual expressions of fear

In their drawings, children often exaggerated the size of needles and syringes. An 11-year-old girl (P3) explained her drawing (see Fig. 2a): *“I drew the syringe big because it felt scary”*, visually amplifying her anticipatory anxiety. Similarly, an 11-year-old boy (P12) stated of his drawing (see Fig. 2b), *“I made it huge and black, resembling a giant bee sting”*. This fear of the procedural act was equally evident in the narrative of a 10-year-old boy, who drew a detailed depiction of the needle entering his arm and accompanied it with the explanation, *“I felt the needle go in, and it was scary”*—demonstrating how the moment of needle insertion constituted the focal point of his fearful experience, both visually and emotionally.

##### Subtheme 1.3: Positive psychological outcomes

Despite the challenges associated with medical procedures, children report positive feelings and outcomes from the experience. This demonstrates how positive psychological framing can influence their overall perception and help them find beneficial aspects even in difficult situations. These positive outcomes often include feelings of pride, relief, or a sense of accomplishment, which can contribute to their emotional resilience and confidence.

**Fig. 3:**
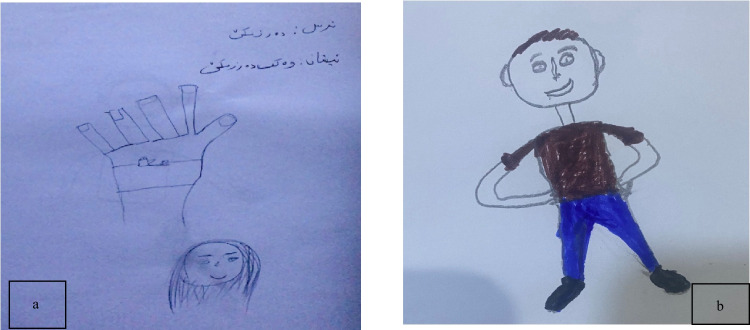
Drawings **a** and **b** illustrate children’s expressions of positive psychological outcomes

Despite procedural distress, children reported feelings of pride and relief post-procedure. A 10-year-old boy (P10) articulated his pride, stating, *“I felt proud of myself for being brave”* (see Fig. 3a). Concurrently, a 12-year-old girl (P4) visually emphasized her emotional relief by drawing a happy face after the injection (see Fig. 3b).

#### Theme 2: Parental support as a psychological buffer

In this theme, the children expressed the crucial role of parental support in mitigating their psychological distress during venipuncture. The presence and involvement of parents acted as a psychological buffer, providing comfort, reassurance, and a sense of security. The significance of this buffer becomes particularly apparent when considered alongside the feelings of spatial vulnerability expressed in several children’s drawings: three children combined both the hospital setting and the venipuncture site in their artwork, using spatial arrangements that—according to their accompanying narratives—conveyed a profound sense of isolation. One child explained that drawing themselves in the center of a large room made them feel “very small and alone in the big room”. It is precisely against this backdrop of felt smallness and institutional vastness that parental presence emerges not merely as comforting, but as psychologically indispensable. The children highlighted how the physical and emotional support from their parents—such as holding their hands or offering hugs—helped them feel calmer and less fearful during the procedure. This theme underscores the importance of parental presence in creating a safe and supportive environment for children during venipuncture experiences.

##### Subtheme 2.1: Parental holding and comfort

Children expressed significant comfort in having their parents hold their hands or hug them during medical procedures. This physical contact provides a sense of security and helps reduce feelings of fear and anxiety, making the experience less overwhelming and more manageable for the child. The warmth and closeness of a parent’s touch offer tactile reassurance, helping children feel supported, protected, and less alone during stressful moments. This simple yet powerful act of holding hands or offering a hug can create a calming effect, allowing children to feel more grounded and confident as they face challenging situations. By providing this physical comfort, parents play a vital role in easing their child’s emotional distress and fostering a sense of safety during medical procedures.

**Fig. 4:**
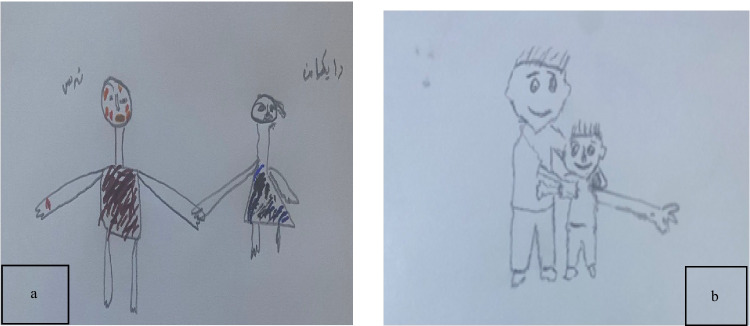
Drawings **a** and **b** illustrate the concept of parental comfort

Children expressed significant comfort in this physical contact. A 6-year-old boy (P14) said, *“My dad’s hug made me feel better and less scared” *(see Fig. 4a). Similarly, an 8-year-old boy (P5) noted,* “When my mom held my hand, I felt calmer”* (see Fig. 4b).

##### Subtheme 2.2: Security and reassurance

Children expressed that having their parents present during medical procedures provided them with a strong sense of security and reassurance. The presence of a parent or caregiver helped alleviate their anxiety and fear, making the experience more manageable. Knowing that a trusted adult was by their side gave them the courage to face the procedure and reduced feelings of loneliness or helplessness. This emotional support not only comforted them but also helped them feel more in control during a potentially stressful situation.

**Fig. 5:**
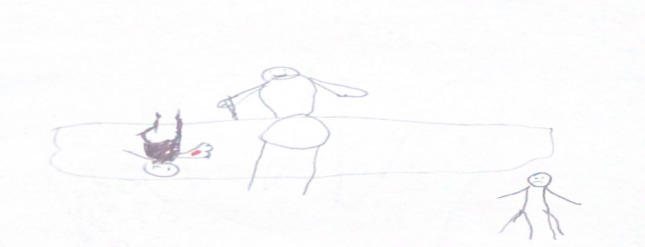
Drawing a parent’s reassuring presence during the procedure

This emotional security was frequently described. A 9-year-old girl (P6) captured this sentiment, noting, *“Having my mom by my side made me feel less scared”* (see Fig. 5), highlighting how parental presence directly reduced feelings of fear and loneliness.

#### Theme 3: Ease and comfort

Children expressed how preparation, distraction techniques, and praise and encouragement from caregivers and healthcare providers helped them feel at ease and comfortable during venipuncture. These strategies reduced fear and anxiety, creating a sense of safety and reassurance. By knowing what to expect, engaging in distractions, and receiving positive reinforcement, children felt empowered to face the procedure with greater confidence.

##### Subtheme 3.1: Preparation and information

Children expressed that knowing what to expect during a venipuncture procedure, like getting a shot or having blood drawn, helped them feel more in control and less scared. When caregivers or nurses took the time to explain the steps of the procedure and describe what they might feel—such as a quick pinch or pressure—it made the whole experience easier to handle. Understanding what was going to happen helped them feel prepared and less surprised, which made them feel braver and more confident. This kind of clear and honest communication turned something scary into something they could manage, showing how important it is to give children the information they need to feel safe and supported.

**Fig. 6:**
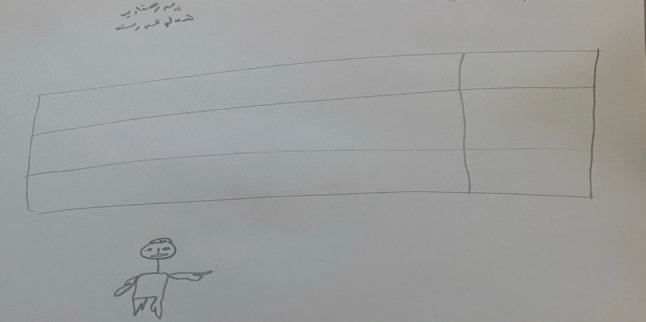
Drawing of a nurse providing a procedural explanation

This value of preparation was reflected in both narrative and visual data. A 10-year-old girl (P7) recalled, *“The nurse explained what would happen”*, and her accompanying drawing (see Fig. 6) visually represented this moment of explanation, illustrating how procedural information fostered predictability and reduced anxiety.

##### Subtheme 3.2: Distraction techniques

Children described a variety of distraction and coping techniques that helped them manage their psychological responses to medical procedures. These strategies included actions like closing their eyes, looking away, or engaging in non-procedural conversation with caregivers or nurses. By focusing their attention on something other than the procedure itself, these techniques effectively diverted their thoughts away from the pain and fear they might otherwise feel. This shift in focus made the experience more manageable and less overwhelming, allowing them to feel more in control and less anxious.

**Fig. 7:**
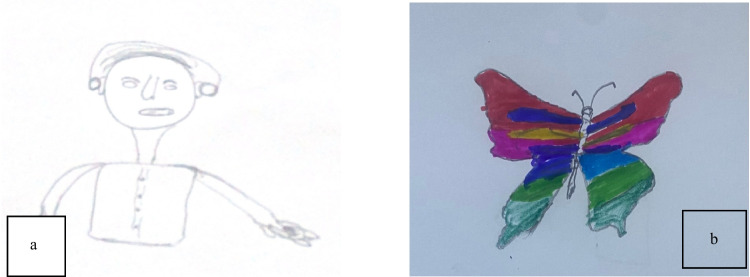
Drawings **a** and **b**: children’s visual representations of distraction strategies

Children described using specific, cognitive methods to shift their focus. A 7-year-old boy (P8) shared, *“I closed my eyes and contemplated the music from my favorite cartoon”* (see Fig. 7a). Similarly, a 6-year-old girl (P13) explained her strategy, stating, *“I simply focused on the sticker on the wall and counted its colors”* (see Fig. 7b). These participant-initiated distraction techniques effectively diverted attention from the procedural pain and fear.

##### Subtheme 3.3: Praise and encouragement

Children expressed that supportive communication from caregivers or nurses, including praise and encouragement, was essential in helping them manage the emotional and psychological challenges of venipuncture procedures, such as injections. They valued being praised for their bravery or cooperation after the procedure, with some explicitly stating they needed such positive reinforcement. Encouraging words during the procedure also provided comfort, motivation, and distraction. This theme underscores the importance of personalized, supportive communication in fostering safety, trust, and confidence, making challenging medical experiences more manageable and less stressful.

**Fig. 8:**
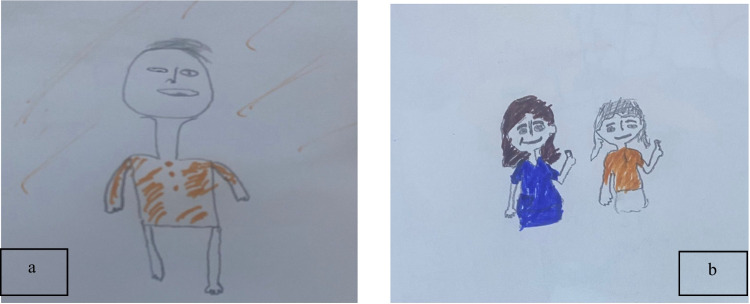
Drawings **a** and **b**: visual representations of receiving praise

This supportive communication was described as highly impactful. A 9-year-old boy (P9(2)) stated, *“The nurse said I was brave, and it made me feel stronger”* (see Fig. 8a). This experience was echoed by a 7-year-old girl (P11), who recalled, *“After it was done, the nurse told me I did a great job”* (see Fig. 8b). This positive reinforcement from healthcare providers was a key factor in reinforcing children’s self-efficacy and coping.

## Discussion

This qualitative descriptive study used the Drawing-and-Storytelling (DS) method to explore the psychological experiences of Kurdish children (aged 6–12 years) undergoing venipuncture in a tertiary hospital in the Kurdistan Region of Iraq. The findings, structured around the themes of Pain and Fear, Parental Support as a Psychological Buffer, and Ease and Comfort Strategies, provide a nuanced, child-centered account of procedural distress within a specific cultural and healthcare context. This analysis, grounded in the participants’ own narratives and visual data, offers an interpretative account of their lived experiences[31]. The following discussion interprets these findings through the integrated lenses of Child-Centered Care (CCC) and Family Systems Theory (FST), linking them to existing evidence and highlighting their practical significance.

While the broader practice model of Family-Centered Care (FCC) advocates for involving parents, our use of Family Systems Theory (FST) provides a deeper, mechanistic understanding of the dynamics observed[13]. FCC outlines the principle of family inclusion, whereas FST illuminates the process of how the family functions as an interconnected emotional unit under stress. This theoretical lens moves us beyond simply noting that parents were present to analyzing the reciprocal, regulatory interactions that constitute “support”. It positions the parent–child dynamic not as a simple resource but as an active, bidirectional process central to the child’s lived experience, thereby offering a more nuanced explanatory framework than FCC alone.

### Pain and fear during venipuncture: subjectivity and anticipation

Children’s vivid expressions of pain and fear, often amplified in their drawings (e.g. exaggerated needle sizes, use of dark colours), underscore the highly subjective and distressing nature of venipuncture. This aligns with existing literature where needle fear and anticipatory anxiety are significant drivers of procedural distress, sometimes exceeding the pain itself [1, 34]. Our findings extend this understanding by capturing, through the children’s own visual and verbal narratives, how imagination magnifies these fears[35]. Thematic analysis of this multimodal data allowed these subjective perceptions to emerge as central to their experience. The reported positive psychological outcomes—feelings of pride and relief post-procedure—are crucial. They demonstrate that a distressing event can be reframed, resonating with research on coping and resilience[1]. This underscores a core tenet of CCC: by acknowledging and validating both the fear and the subsequent achievement, healthcare providers can help shape a more empowering narrative for the child [8].

### Parental support as a psychological buffer: a systemic dynamic

The present findings corroborate the well-established importance of parental involvement in paediatric procedural pain management [13, 36, 37]. Parental presence, physical contact, and emotional reassurance consistently emerged as the primary sources of psychological mitigation during venipuncture. In this analysis, the term holding is used in a dual sense: at the behavioural level, it refers to the concrete parental acts of hand-holding and physical embracing reported by the children as immediate sources of tactile comfort and reassurance; at the relational level, it draws upon Winnicott [38] concept of the holding environment—the caregiver’s capacity to provide a psychologically containing presence that sustains the child’s affective equilibrium under acute stress [38, 39].

Viewed through an FST lens, the children’s descriptions of feeling “calmer” when held illustrate a process of dyadic affect regulation within the family system. The parent’s capacity to manage their own anxiety and the child’s proximity-seeking together constitute a reciprocal, stabilizing feedback loop—a bidirectional regulatory process in which the parent functions as an active co-regulator rather than a passive comforting agent [13, 40]. This systemic perspective moves beyond simply noting parental presence to explaining how the family unit organizes itself to buffer procedural stress, highlighting that supporting the parent–child dyad is as clinically critical as supporting the child alone. This is particularly salient in the studied context, where close-knit family structures and constraints on nursing time may naturally elevate the parent's role as the primary psychological buffer.

### Ease and comfort strategies: the practical application of CCC

The children identified preparation, distraction, and praise as key strategies fostering ease. These are well-supported, evidence-based interventions [6, 41]. Their significance in this study lies in how they were *valued and described by the children themselves*, validating their relevance in this specific setting. For instance, a child recalling that “the nurse explained what would happen” highlights how simple, honest communication fulfills the CCC principle of respect for the child’s need for information and control [8, 42]. Similarly, self-initiated distraction (e.g. thinking of a favorite song) and the valuing of praise for bravery represent active coping and positive reinforcement, aligning with CCC’s goal of empowering the child as an active participant in their care. These participant-identified strategies provide emic validity to established clinical guidelines.

### Strengths, limitations, and reflexivity

A primary strength of this study is its child-centered, arts-based methodology, which facilitated deep engagement and rich, multimodal data generation, empowering participants as active co-constructors of meaning[43]. The use of reflexive thematic analysis allowed for a nuanced interpretation of patterns across both verbal and visual data[32, 44]. Limitations that affect the transferability of findings include the use of consecutive sampling from a single tertiary-care site and a modest sample size, though the latter is consistent with the depth-oriented goals of qualitative research. The lack of formal member-checking post-analysis, despite in-session verbal confirmations, and potential recall bias are also acknowledged. The research team’s reflexive awareness of their insider/outsider positions—with a Kurdish-speaking clinician-researcher conducting interviews and an international supervisory team guiding analysis—served to balance cultural intimacy with methodological rigour.

### Implications for practice

The findings suggest several implications for clinical practice in similar contexts: (1) formally integrate and support parental presence during procedures, recognizing their role as active agents within the care system; (2) implement structured, child-led comfort protocols that prioritize developmentally appropriate preparation and a menu of distraction techniques; and (3) train healthcare providers in communication skills that employ praise and positive framing to reinforce coping.

### Future directions

Future research should (1) include the perspectives of parents and nurses in this context to triangulate findings; (2) develop and test interventions based on DS-informed insights, such as pre-procedure drawing activities; (3) conduct cross-cultural comparisons to distinguish universal from context-specific factors; and (4) undertake longitudinal studies to understand the long-term impact of procedural distress in similar settings.

## Conclusion

In conclusion, this study used an arts-based, child-centered method to illuminate the psychological landscape of venipuncture for Kurdish children. It confirms the universality of pain and fear while highlighting the profound, culturally situated buffering role of the family system, understood as a dynamic relational unit. The findings advocate for a dual-focused clinical approach: implementing practical, child-validated comfort strategies while concurrently nurturing the supportive capacity of the parent–child dyad. Ultimately, integrating the child’s subjective perspective with a systemic understanding of family support is paramount for advancing psychological care during invasive paediatric procedures.

## Data Availability

The datasets analysed during the current study are available from the corresponding author upon reasonable request.
